# Unveiling the Potential Role of Glucagon-Like Peptide-1 (GLP-1) Receptor Agonists in Offering Protection of the Cardiovascular, Renal, and Neural Systems: An Updated Narrative Review

**DOI:** 10.7759/cureus.65910

**Published:** 2024-07-31

**Authors:** Divya Rajagopal, Sulthan Al Rashid, Monisha Prasad, Mohammad Fareed

**Affiliations:** 1 Department of Pharmacology, Saveetha Medical College and Hospital, Saveetha Institute of Medical and Technical Sciences (SIMATS), Saveetha University, Chennai, IND; 2 Center for Global Health Research, Saveetha Medical College and Hospital, Saveetha Institute of Medical and Technical Sciences (SIMATS), Saveetha University, Chennai, IND

**Keywords:** neuroprotective effects, renoprotective effects, cardioprotective effects, type 2 diabetes mellitus, glp-1 receptor agonists

## Abstract

Glucagon-like peptide-1 (GLP-1) receptor agonists have drawn a lot of interest lately for their therapeutic advantages over controlling blood sugar levels in the management of type 2 diabetes mellitus (T2DM). This review aims to provide an overview of the research that has been done on the neuroprotective, renoprotective, and cardioprotective effects of GLP-1 receptor agonists. Studies suggest that these medicines could provide protective benefits beyond glucose regulation, possibly reducing the risks of cardiovascular and renal issues; mechanisms underlying these advantages are still not fully understood. The review emphasizes how crucial it is to conduct more studies to determine the clinical significance and underlying mechanisms of these protective benefits. Improved knowledge of GLP-1 receptor agonists may result in T2DM treatment plans that improve neurological, cardiovascular, and renal function in addition to blood sugar control. Therefore, further research is necessary to fully understand the potential of GLP-1 receptor agonists in providing comprehensive protection against complications related to T2DM.

## Introduction and background

Diabetes mellitus is a long-standing metabolic ailment distinguished by heightened blood sugar levels. This condition arises when the body fails to either generate adequate insulin or properly utilize the insulin it produces [1]. The worldwide incidence of diabetes has been on the rise, presenting notable health hurdles. As per the World Health Organization (WHO), the global occurrence of diabetes was approximately 8.5% in 2019, impacting approximately 463 million people [2]. The burden of diabetes in India is notable, with an estimated 87 million adults diagnosed with the condition in 2019, according to the International Diabetes Federation (IDF). By 2045, this number is anticipated to increase to 151 million, making India the country with the largest diabetic population worldwide. The prevalence of diabetes among Indian adults aged 20-79 years is also alarming, affecting around 11.8% of this demographic [3]. The significant prevalence of diabetes in India has various ramifications for public health, straining healthcare systems in terms of both diagnosing and treating the condition. Furthermore, complications associated with diabetes, including cardiovascular issues, kidney diseases, and eye ailments, lead to heightened levels of illness and death [4].

Diabetes mellitus is linked with notable cardiovascular and renal issues. Diabetic nephropathy, a prevalent renal complication, is marked by gradual kidney deterioration resulting in decreased kidney performance. It represents a primary factor contributing to end-stage renal disease [5]. Cardiovascular complications arising from diabetes encompass conditions such as coronary artery disease, stroke, peripheral artery disease, and heart failure [6]. These complications are responsible for increased morbidity and mortality in individuals with diabetes.

Few medications for diabetes have been shown to not only lower blood sugar but also protect the heart and kidneys. Sodium-glucose co-transporter 2 (SGLT-2) inhibitors, for instance, have been proven to decrease cardiovascular incidents, reduce hospitalizations due to heart failure, and enhance kidney health in people with type 2 diabetes mellitus (T2DM) [7]. Glucagon-like peptide-1 (GLP-1) receptor agonists, a newly developed category of medications for diabetes management, have demonstrated potential cardioprotective, nephroprotective, and also neuroprotective effects in individuals with T2DM. These medications have demonstrated effectiveness in lowering the chances of severe cardiovascular events, including heart-related deaths, nonfatal heart attacks, and strokes (neuroprotective) [8]. Moreover, GLP-1 receptor agonists have been linked to enhanced kidney health, characterized by decreased levels of albumin in the urine and a potential deceleration in the advancement of chronic kidney disease (CKD) [9].

In addition to β-cell dysfunction, the absence of incretins is now recognized as one of the significant contributors to the development of T2DM [10]. Consequently, GLP-1 receptor agonists have emerged as a promising therapeutic option for managing diabetes. Thus, this review explores the recent evidence-based studies regarding the potential clinical advantages of these agonists for their potential in offering protection pertaining to cardiovascular, renal, and neurovascular systems.

## Review

Methodology

The online databases, including MEDLINE/Pubmed/PubMed Central® (PMC), Google Scholar, ScienceDirect, EBSCO, Scopus, Web of Science, Embase, and reference lists, were thoroughly searched using specific keywords such as GLP-1 receptor agonists (e.g., exenatide, lixisenatide, liraglutide, dulaglutide, albiglutide, semaglutide, tirzepatide), T2DM, as well as terms related to cardioprotection, renoprotection, and neuroprotection. Only publications written in English that discussed the cardioprotective, renoprotective, and neuroprotective effects of GLP -1 receptor agonists were considered, while duplicate entries were removed from the analysis.

Pharmacology of GLP-1 Receptor Agonists

The pharmacokinetic properties of all GLP-1 receptor agonist drugs are discussed below (Tables 1-2):

**Table 1:** Different GLP-1 receptor agonists drugs, their dose and frequency GLP-1: Glucagon-like peptide-1

GLP-1 Agonists		
Drug	Dose (mg)	Dose Frequency
Dulaglutide	0.75–4.5	Weekly
Exenatide	0.005–0.010	Daily
Exenatide, extended release	2	Weekly
Liraglutide	1.2–1.8	Daily
Lixisenatide	0.010–0.020	Daily
Semaglutide	0.5–1.0	Weekly
Semaglutide, oral	7–14	Daily

**Table 2:** Pipeline drugs of GLP-1 agonist receptors with their recommended dose GLP-1: Glucagon-like peptide-1

Drug Name	Dose
Oral Semaglutide	50 mg OD
Tirzepatide	5/10/15 mg OD
Orfloglipron	12/24/36/45 mg OD
Carglinitide	0.3/0.6/1.2/2.4/4.5 mg OD
Survodutide	0.6/2.4/3.6/4.8 mg OD
Efinopeglutide	5/7.4/10 mg OD
Mazdutide	9 mg Od
Pemvidutide	1.2/1.8.2.4 mg Od
Retatrutide	1/4/8/12 mg Od
Cagrinilitide Plus Semaglutide	0.16/0.3/0.6/1.2/2.4/4.5 mg Plus sema 2.4 mg

Albiglutide: With a half-life spanning around four to seven days, albiglutide permits weekly administration due to its extended duration of action. It undergoes breakdown by proteolytic enzymes and is eliminated through the kidneys. Albiglutide has demonstrated comparable effectiveness to other GLP-1 receptor agonists concerning managing blood sugar levels and promoting weight loss [11].

Dulaglutide: Dulaglutide's half-life ranges from four to five days, enabling weekly administration due to its prolonged duration of action. It undergoes degradation via proteolysis and is eliminated through the kidneys. Studies have demonstrated that dulaglutide is as effective as daily liraglutide in managing blood sugar levels and cardiovascular outcomes [12].

Exenatide: Exenatide exhibits a half-life of around 2.4 hours, necessitating dosing twice a day. It is primarily cleared through the kidneys, with minimal metabolic processing by the liver. Research indicates that exenatide not only enhances glycemic regulation but also contributes to weight reduction [13].

Liraglutide: Liraglutide possesses a prolonged half-life of roughly 13 hours, permitting administration once a day. Its main route of elimination is through the kidneys, and its pharmacokinetics remain largely unaffected by age or renal dysfunction. Additionally, liraglutide has been demonstrated to be as effective as twice-daily exenatide in managing blood sugar levels [14].

Lixisenatide: Lixisenatide needs to be taken once daily due to its short half-life of around 2.5 hours. It is primarily cleared from the body through the kidneys with minimal involvement of the liver. Studies have demonstrated that lixisenatide effectively enhances glycemic management and decreases elevated blood sugar levels after meals [15].

Semaglutide: Semaglutide, with a half-life of roughly seven days, permits weekly dosing. It is broken down by proteolytic processes and eliminated through the kidneys. Additionally, semaglutide has demonstrated superior efficacy compared to other GLP-1 receptor agonists in lowering the risk of cardiovascular events and enhancing glycemic management [16]. Oral semaglutide, with a half-life of about one week, permits daily administration. It is absorbed extensively in the gastrointestinal tract, followed by breakdown through proteolytic processes and elimination via the kidneys. Oral semaglutide has demonstrated effectiveness in enhancing glycemic regulation and cardiovascular outcomes comparable to injectable GLP-1 receptor agonists [17].

Tirzepatide: Tirzepatide acts as both a GIP and GLP-1 receptor agonist simultaneously. Tirzepatide, with a half-life of around 2-3 days, permits weekly dosing. It is broken down through proteolytic processes and eliminated via the kidneys. Clinical trials have shown that tirzepatide offers better glycemic management and weight loss compared to other GLP-1 receptor agonists [18].

Mechanism of Action of GLP-1 Receptor Agonists

GLP-1 receptor agonist medications replicate the effects of natural GLP-1, a hormone discharged by the intestines following food consumption. By binding to and stimulating GLP-1 receptors, these agonists prompt insulin secretion by pancreatic beta cells, inhibit glucagon release, delay stomach emptying, and increase feelings of fullness. These functions aid in enhancing glucose regulation, lowering both fasting and post-meal blood sugar levels, and facilitating weight loss in people diagnosed with T2DM [19].

Potential Mechanisms for the Cardioprotective Actions of GLP-1 Receptor Agonists

Improved cardiac function: GLP-1 receptor agonists have demonstrated benefits for heart health by boosting the uptake of glucose by heart muscle cells, lowering oxidative stress levels, and raising nitric oxide production. These effects result in enhanced heart muscle function and a decreased likelihood of experiencing negative cardiovascular events [20].

Anti-inflammatory and anti-atherosclerotic properties: GLP-1 receptor agonists display strong anti-inflammatory properties, alleviating the persistent low-grade systemic inflammation linked to cardiovascular illness. Furthermore, they decrease the development of atherosclerotic plaques and encourage their stability, consequently diminishing the likelihood of cardiovascular incidents [20].

**Figure 1:**
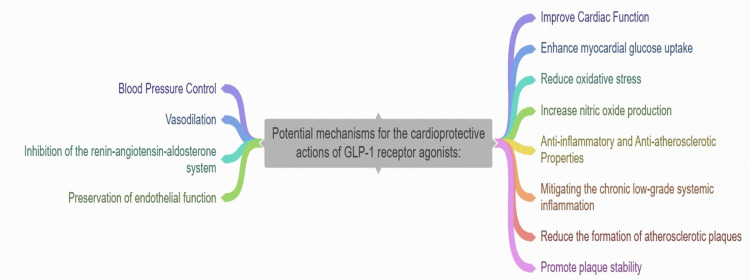
Different mechanisms for the cardioprotective actions of GLP-1 receptor agonists GLP-1: Glucagon-like peptide-1 This figure was created by the authors

Blood pressure control: GLP-1 receptor agonists have been discovered to decrease blood pressure through different means, such as vasodilation, restraining the renin-angiotensin-aldosterone system, and maintaining endothelial function. These actions lead to improved cardiovascular results for individuals with hypertension (Figure 1) [21].

Potential Mechanisms for the Nephroprotective Actions of GLP-1 Receptor Agonists

GLP-1 receptor agonists have demonstrated encouraging protective effects on the kidneys in both preclinical and clinical studies. Some of the possible ways they might deliver these protective effects include the following:

Anti-inflammatory effects: GLP-1 receptor agonists have been shown to diminish kidney inflammation by suppressing the production of pro-inflammatory molecules and inhibiting the activation of nuclear factor-kappa B (NF-κB), a central mediator of inflammation [8].

Oxidative stress reduction: GLP-1 receptor agonists may additionally aid in diminishing oxidative stress within the kidneys. They have the capability to boost the function of antioxidant enzymes like superoxide dismutase (SOD) and catalase while suppressing the generation of reactive oxygen species (ROS) [21].

**Figure 2:**
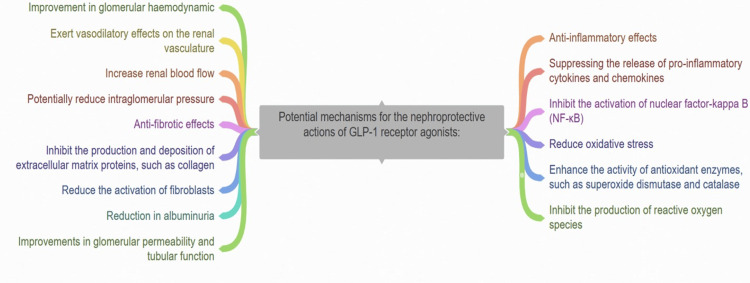
Different mechanisms for the nephroprotective actions of GLP-1 receptor agonists GLP-1: Glucagon-like peptide-1 This figure was created by the authors

Improvement in glomerular hemodynamic and reduction in albuminuria: GLP-1 receptor agonists have demonstrated the ability to induce vasodilation in the renal blood vessels. This action can enhance glomerular hemodynamics, elevate renal blood flow, and possibly lower intraglomerular pressure, offering advantages for individuals with renal dysfunction [22]. GLP-1 receptor agonists have been noted to lower albuminuria, a significant marker of kidney damage and progression of renal disease risk. Although the exact mechanism is not completely understood, this effect might involve improvements in both glomerular permeability and tubular function (Figure 2) [23].

Anti-fibrotic effects: GLP-1 receptor agonists have the potential to alleviate renal fibrosis, a prevalent pathological condition in CKD. They can hinder the synthesis and accumulation of extracellular matrix proteins like collagen and diminish the activation of fibroblasts [24].

Potential Mechanisms for Decreasing the Risk of Stroke with GLP-1 Receptor Agonists

**Figure 3:**
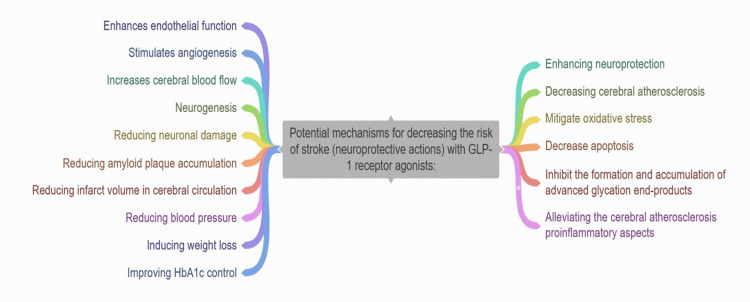
Different mechanisms for decreasing the risk of stroke with GLP-1 receptor agonists This figure was created by the authors

Multiple pathways contribute to the cardiovascular, metabolic, and renal advantages of GLP-1 receptor agonists [25]. These drugs are thought to lower the risk of stroke by enhancing neuroprotection and diminishing cerebral atherosclerosis [26]. They mitigate oxidative stress, reduce cell death, and hinder the production and buildup of advanced glycation end-products, thus alleviating the cerebral atherosclerosis proinflammatory aspects [27]. Additionally, GLP-1 receptor agonists therapy enhances endothelial function, promotes the growth of new blood vessels, boosts blood flow to the brain, and fosters the development of new neurons while also reducing damage to nerve cells, accumulation of amyloid plaques, and the volume of tissue damage in the brain's blood circulation [28]. Indirectly, GLP-1 receptor agonists decrease stroke risk by lowering blood pressure, inducing weight loss, and improving HbA1c. Notably, in an analysis, it was discovered that the decrease in HbA1c levels accounted for a substantial portion (54%) of the stroke risk reduction observed in the "Dulaglutide and cardiovascular outcomes in T2DM" (REWIND) trial [29]. Moreover, a meta-regression analysis indicates a direct association between the reduction in HbA1c levels and the decrease in stroke risk (Figure 3) [30].

Safety Profile

The use of GLP-1 receptor agonists can lead to various adverse effects including gallstones, increased heart rate, retinopathy, medullary thyroid cancer, and pancreatitis. In the "Liraglutide Effect and Action in T2DM: Evaluation of Cardiovascular Outcome Results" (LEADER) trial, liraglutide was associated with a higher incidence of acute gallstones compared to placebo [31]. Similar findings were reported in studies with exenatide and liraglutide [32]. The "Semaglutide and Cardiovascular Outcomes in Patients with T2DM" (SUSTAIN 6) trial showed that diabetic retinopathy occurred in 3% of patients treated with semaglutide, with a similar but nonsignificant trend observed in the LEADER trial. However, a meta-analysis involving over 2000 participants did not confirm these results [33]. Notably, large population studies and recent meta-analyses have not reported occurrences of pancreatitis or medullary thyroid carcinoma [17].

The mechanism behind the increased heart rate associated with GLP-1 receptor agonists is not fully understood but may involve direct action on the sinoatrial node. This effect could have negative implications for patients with heart failure. Both liraglutide (LEADER trial) and semaglutide (SUSTAIN 6 trial) showed a nonsignificant increase in heart rate [34]. Consequently, SGLT-2 inhibitors are preferred in T2DM patients with heart failure due to their more favorable outcomes.

Trials on Cardioprotective Effects of GLP-1 Receptor Agonists

Numerous studies have explored the cardiovascular benefits of GLP-1 agonists in patients with either existing cardiovascular disease or a heightened risk of cardiovascular issues. Noteworthy trials in this regard include the following: 

The LEADER trial: This study evaluated the cardiovascular effects of liraglutide in individuals diagnosed with T2DM and at a heightened risk of cardiovascular complications. It demonstrated a decrease in major adverse cardiovascular events, such as cardiovascular mortality, nonfatal myocardial infarction, and stroke, when compared to a placebo [34].

The SUSTAIN 6 trial: The trial examined the cardiovascular safety and effectiveness of semaglutide in individuals with T2DM who were at a high risk of cardiovascular complications. Semaglutide exhibited a decrease in major adverse cardiovascular events compared to a placebo [33].

The "Harmony Outcomes" trial: This research examined the effects of albiglutide on cardiovascular health in individuals with T2DM and existing cardiovascular issues. While albiglutide didn't prove to be significantly better than a placebo in lowering major adverse cardiovascular events, it did demonstrate comparable effectiveness, meeting the criteria for non-inferiority [30].

Dulaglutide (Trulicity): The REWIND trial revealed that dulaglutide decreased the likelihood of major adverse cardiovascular events (MACEs), cardiovascular mortality, and a combined renal outcome in patients diagnosed with T2DM and cardiovascular risk factors when compared to a placebo [30].

Exenatide extended-release (bydureon): In the EXSCEL trial, exenatide extended-release did not demonstrate a notable decrease in MACEs when contrasted with a placebo. However, it did meet the standards for non-inferiority concerning cardiovascular safety in individuals diagnosed with T2DM and at high risk for cardiovascular issues [30].

Trials on the Nephroprotective Effect of GLP-1 Receptor Agonists

The LEADER trial: This study evaluated the cardiovascular and renal outcomes of the GLP-1 receptor agonist liraglutide in patients with T2DM. The study showed that liraglutide reduced the risk of new-onset macroalbuminuria and progression of existing renal disease compared to placebo [31].

The REWIND trial: This study investigated the cardiovascular and renal effects of the GLP-1 receptor agonist dulaglutide in patients with T2DM. The trial found a significant reduction in the composite outcome of new or worsening nephropathy with dulaglutide compared to placebo [32].

Meta-analysis Reports of GLP-1 Receptor Agonists and Other Antidiabetic Medications in Preventing Stroke

A meta-analysis encompassing seven cardiovascular outcome trials (CVOTs) with approximately 56,000 patients diagnosed with T2DM found that the use of GLP-1 receptor agonists was associated with a 15% decrease in the incidence of nonfatal stroke [35]. Among 48 randomized studies covering eight classes of antihyperglycemic treatments and reporting stroke outcomes, only GLP-1 receptor agonists and thiazolidinediones exhibited statistically significant reductions in stroke occurrences [36]. SGLT-2 inhibitors were found to reduce kidney endpoints, hospitalizations for heart failure, and the composite of MACEs across various patients with T2DM; however, they did not demonstrate a reduction in stroke incidence [35,36]. 

American Association of Clinical Endocrinology (AACE) Clinical Practice Guideline Related to GLP-1 Analogues and Stroke

Utilize GLP-1 receptor agonists in individuals diagnosed with T2DM and either established atherosclerotic cardiovascular disease (ASCVD) or at a heightened risk for ASCVD to mitigate the likelihood of myocardial infarction, stroke, or cardiovascular death, irrespective of A1C levels and other therapies targeting glucose or cardiovascular health. Administer GLP-1 receptor agonists to individuals with T2DM and either existing ASCVD or those predisposed to ASCVD, as these agents have demonstrated efficacy in lowering the occurrence of stroke [37].

## Conclusions

In conclusion, the cardiorenal and neuroprotective characteristics of GLP-1 receptor agonists highlight their significant role in managing individuals newly diagnosed with T2DM and hypertension. These patients face an increased risk of developing cardiovascular and renal complications such as ASCVD and CKD. While SGLT-2 inhibitors also demonstrate cardiorenal protective properties, they do not appear to offer protection against stroke, as indicated by various meta-analytical studies. Given that T2DM, hypertension, and atrial fibrillation (AF) are critical risk factors for stroke, it is crucial to consider the implications for patients diagnosed with all three conditions simultaneously.

Therefore, prioritizing GLP-1 receptor agonists over SGLT-2 inhibitors and other antidiabetic medications is recommended in such scenarios. This preference is due to the comprehensive cardiorenal effects, including antihypertensive attributes, and their neuroprotective mechanisms against stroke. Further research is needed to validate the full scope of GLP-1 agonists' potential in managing these conditions effectively and comprehensively.

Additionally, exploring and evaluating the neuroprotective effects of GLP-1 agonists in conditions with elevated stroke risk factors should be a priority for future investigations. Understanding these mechanisms could lead to tailored treatment strategies that address both metabolic and neurological aspects of patients' health.

Looking forward, the implications of incorporating GLP-1 receptor agonists into clinical practice extend beyond managing traditional cardiovascular and renal risks. They offer promising avenues for reducing the burden of stroke and other neurological complications in patients with complex cardiometabolic conditions. Future studies should continue to explore these multifaceted benefits to optimize therapeutic outcomes and enhance patient care.
